# A Multicellular 3D GelMA‐Based Colorectal Cancer Model for Chemotherapeutic Responses

**DOI:** 10.1002/mabi.70190

**Published:** 2026-05-01

**Authors:** Seyfure Adıgüzel, Sevde Altuntaş, Şevval Çelikten, Mehmet Cem Gündüz, Merve Güdül Bacanlı

**Affiliations:** ^1^ Department of Molecular Biology and Genetics University of Health Sciences Turkey Istanbul Türkiye; ^2^ Experimental Medicine Research and Application Center University of Health Sciences Turkey Istanbul Türkiye; ^3^ Department of Tissue Engineering Institution of Health Sciences University of Health Sciences Turkey Istanbul Türkiye; ^4^ Gülhane Faculty of Pharmacy Department of Pharmaceutical Toxicology University of Health Sciences Turkey Ankara Türkiye; ^5^ Graduate Program of Biomedical Engineering Koc University Istanbul Türkiye; ^6^ Turkish Vaccine Institute Health Institutes of Türkiye (TÜSEB) Aziz Sancar Research Center Ankara Türkiye

**Keywords:** 3D cell culture, colorectal cancer, drug screening, GelMA hydrogel, in vitro modeling, tumor microenvironment

## Abstract

Advanced biomaterials‐based *in vitro* platforms are increasingly required to overcome the limited predictive power of conventional 2D cell cultures in colorectal cancer (CRC) drug screening. Herein, we report the development of a biomimetic, multicellular 3D CRC model based on gelatin methacrylate (GelMA) hydrogels, designed to recapitulate key structural and biological features of the tumor microenvironment. The platform integrates a vascularized hydrogel compartment with human endothelial cells, combined with cancer‐associated fibroblasts and macrophages, enabling controlled tumor‐stroma‐vessel interactions within a physiologically relevant architecture. The GelMA hydrogels were comprehensively characterized, and their role in regulating endothelial viability, migration, and angiogenic marker expression was systematically evaluated. The drug screening capability of the platform was assessed using 5‐fluorouracil (5‐FU). Comparative analyses revealed that 3D cultures exhibited attenuated cytotoxicity, oxidative stress, and apoptotic responses relative to 2D monolayers, particularly at lower drug concentrations and prolonged exposure times. These findings demonstrate that GelMA‐based microenvironment actively modulates cellular drug responses through multicellular interactions and diffusion‐mediated effects, rather than acting as a passive scaffold. Overall, this study establishes a functional 3D *in vitro* platform that provides improved physiological relevance and predictive capability for preclinical CRC drug screening, while offering a human‐relevant alternative aligned with the principles of the 3Rs.

## Graphical abstract

The development of a GelMA‐based, multicellular 3D in vitro platform designed to more faithfully recapitulate the colorectal cancer (CRC) tumor microenvironment for drug screening applications. The platform integrates a vascularized hydrogel matrix, cancer‐associated fibroblasts, and M2‐polarized macrophages. The translational relevance of the platform was evaluated using 5‐fluorouracil (5‐FU), a frontline chemotherapeutic agent in CRC treatment.

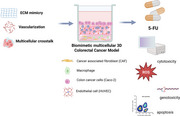

## 1 Introduction

Traditional 2D cell cultures are widely used to assess *in vitro* toxicity and are accepted in preclinical studies, but they poorly predict *in vivo* drug responses [1]. These cultures fail to replicate dynamic *in vivo* tissue environments, forcing cells onto artificial, flat, rigid surfaces that yield inaccurate results. Their limitations in mimicking 3D tissue structures and tumor‐microenvironment interactions are driving the shift to advanced human‐relevant 3D *in vitro* models [2].

Consequently, conventional 2D models inadequately reflect cellular heterogeneity, stromal components, and extracellular matrix (ECM) organization, all of which are critical for understanding tumor biology and therapeutic response. As a result, they often yield limited predictive power in drug screening and contribute to the poor clinical translation of otherwise promising compounds. These limitations have spurred a shift toward sophisticated, human‐relevant 3D *in vitro* models, which offer a more accurate representation of native tissue organization and tumor complexity. Consequently, novel 3D tumor modeling technologies, especially those utilizing hydrogel‐based systems, are designed to overcome the shortcomings of 2D cultures by mimicking ECM properties and cell–matrix interactions. This approach allows for a more precise reproduction of *in vivo* tumor environments, thus facilitating improved predictions of therapeutic efficacy [3].

The development of advanced in vitro models for colorectal cancer (CRC) has become increasingly critical due to the limitations of conventional 2D cell cultures, which fail to accurately recapitulate the complex tumor microenvironment and its physiological relevance. These traditional 2D models often lack the cellular heterogeneity, stromal components, and ECM architecture crucial for understanding tumor biology and drug response [4]. As a result, they yield suboptimal predictability in drug screening and poor translation of promising compounds into clinical success [5]. Accordingly, more physiologically relevant 3D in vitro models are needed to better mimic the intricate *in vivo* tumor environment and provide accurate predictions of therapeutic efficacy [6, 7]. Emerging 3D tumor modeling technologies address these deficiencies by replicating key features of the tumor microenvironment, thereby offering a robust platform for studying cancer progression and therapeutic responses [8].

To address the limitations of traditional 2D systems in CRC research used *in vitro* models, various culture methods are employed. These methods encompass 3D microtissue models, which are produced through diverse culturing techniques, tissue explant models that maintain and propagate colon tumor tissues as layered structures *in vitro*, and air‐liquid interface (ALI) culture models. Microtissue‐based systems facilitate the recreation of cell‐cell and cell‐ECM interactions, thus offering a more physiologically relevant depiction of tumor architecture [6]. Tissue explant models, which preserve the native tissue architecture and heterogeneous cellular composition, facilitate investigations into tumor dynamics and therapeutic responses within an environment that closely approximates the original tumor microenvironment [9]. Furthermore, ALI models promote the development of epithelial cells and intricate structures, thereby offering a valuable approach for examining tumor‐tissue interactions and the specific disease attributes within a controlled laboratory context [10]. Conversely, despite the substantial advantages offered by advanced 3D *in vitro* CRC models in replicating key aspects of the tumor microenvironment, these systems are subject to notable practical limitations. Various 3D culture platforms, such as spheroids, patient‐derived organoids, scaffold‐based constructs, and microfluidic organ‐on‐chip systems, necessitate specialized knowledge and substantial resources for their generation, maintenance, and phenotypic evaluation, which could impede their broader implementation in routine preclinical workflows [11]. Furthermore, while conventional 2D models remain appropriate for automated high‐throughput drug screening assays owing to their scalability and ease of manipulation, modern 3D methodologies often face challenges in high‐throughput compatibility, encompassing issues related to scalability, reproducibility, and assay integration with existing screening platforms [12].

The limitations of current 3D culture systems, along with the shortcomings of 2D models in accurately depicting cellular differences and signaling processes, highlight the growing need for new *in vitro* methods that are more representative of physiological conditions. 3D models that incorporate cancer cells co‐cultured with stromal and vascular cells within hydrogel matrices have garnered considerable interest, given their capacity to more effectively replicate the structural and cellular intricacies of the in vivo tumor microenvironment. Particularly, gelatin methacrylate (GelMA)‐based platforms are crucial for drug screening studies. They closely mimic the natural extracellular matrix. This allows for cell behavior, drug response, and efficacy predictions that are more relevant to the body, compared to traditional 2D culture systems [13]. For instance, hydrogel co‐networks formed from GelMA and poly(ethylene glycol) diacrylate (PEGDA) have demonstrated the ability to facilitate the growth of epithelial cells on their surface, thereby promoting the development of a mature epithelial monolayer. This monolayer closely mirrors critical structural and barrier characteristics of the intestinal mucosa *in vitro*, including physiologically relevant transepithelial electrical resistance and permeability profiles [14].

This study developed a hydrogel‐supported vascular bed compatible with the colorectal cancer tumor microenvironment, established co‐cultures of cancer‐associated fibroblasts (CAF) and macrophages, and created a CRC drug screening model. Hydrogels were synthesized and characterized; the vascular bed was developed with genetic and proteomic analyses of endothelial cells, alongside characterization of CAF/macrophage co‐cultures. For CRC models, CAF/macrophage co‐cultures were seeded on plate substrates, vascular hydrogels were added into the same well, and CRC cells were attached to the hydrogel surface. After optimization, the model's drug screening potential was assessed by evaluating 5‐fluorouracil (5‐FU)’s cytotoxicity, genotoxicity, reactive oxygen species (ROS) production, and apoptosis in 2D human CRC cultures and the developed model.

## 2 Material and Methods

### 2.1 Materials

All chemicals and reagents were of analytical or cell culture grade and used as received unless otherwise stated. Dulbecco's Modified Eagle Medium (DMEM), DMEM/F‐12, RPMI‐1640, phosphate‐buffered saline (PBS), penicillin–streptomycin solution, and 0.25% trypsin–EDTA were obtained from Vivacell (Germany); fetal bovine serum (FBS) from Serena (Germany). The following were from Sigma‐Aldrich (Germany): 3‐(4,5‐dimethylthiazol‐2‐yl)‐2,5‐diphenyltetrazolium bromide (MTT), 2′,7′‐dichlorodihydrofluorescein diacetate (H_2_DCFDA), dimethyl sulfoxide (DMSO), basement membrane extract (BME), normal melting point agarose (NMA), low melting point agarose (LMA), collagenase type II, ethidium bromide (EtBr), ethanol, and sodium hydroxide (NaOH). Enzyme‐linked immunosorbent assay (ELISA) kits for interleukin (IL)‐10 and transforming growth factor (TGF)‐β were from Sunred Biological Technology (China); recombinant human vascular endothelial growth factor (VEGF)‐A, angiopoietin (Ang)‐1, and Ang‐2 from Prospecbio (Israel). Gelatin methacrylate (GelMA) was from ZetaMatrix (Türkiye), and Irgacure 2959 photoinitiator and phorbol 12‐myristate 13‐acetate (PMA) were from Thermo Fisher Scientific (USA). CellTiter‐Glo 3D Cell Viability Assay (Promega, USA). Bicinchoninic acid (BCA) assay kit was obtained from Intron Biotechnology (South Korea). Annexin V/propidium iodide (PI) apoptosis kit was purchased from Serva (Germany).

### 2.2 Apparatus

All experimental procedures were conducted using standardized and calibrated instruments. Thermal properties of the biomaterials were characterized by differential scanning calorimetry (DSC) and thermogravimetric analysis (TGA) using thermal analyzers (PerkinElmer Inc., USA). Fourier‐transform infrared spectroscopy (FT‐IR) measurements were carried out using a spectrometer obtained from Thermo Fisher Scientific (USA). Rheological properties of hydrogel formulations were analyzed using a Kinexus rheometer (Netzsch, Germany). Fluorescence measurements were obtained using a spectrofluorometer (Fluorat‐02 Panorama, Lumex, Canada). Surface morphology of the samples was examined by scanning electron microscopy (SEM) using a Hitachi SU7000 microscope (Hitachi High‐Tech, Japan). UV crosslinking and sterilization procedures were performed using a UV chamber (Vilber Lourmat Biolink, Germany). Cell culture experiments were carried out in standard and hypoxic incubators (Panasonic Healthcare, Japan), as well as in a conventional CO_2_ incubator (Nüve, Türkiye). Gene expression and nucleic acid quantification were performed using a digital PCR system (Bio‐Rad Laboratories, USA). Absorbance measurements for ELISA assays were conducted using a microplate reader (BioTek Instruments, USA). Fluorescence imaging was performed using a fluorescence microscope and a Paula live‐cell imaging system (Leica Microsystems, Germany). DNA damage analysis in comet assay experiments was performed using the Comet Assay IV image analysis system (Perceptive Instruments, UK).

### 2.3 Hydrogel Preparation

GelMA was dissolved in PBS at 3%, 5%, or 7% (w/v) concentrations at 40°C and 500 rpm for 30 min with magnetic stirring. Irgacure 2959 (0.5% v/v) was added as a crosslinker and stirred for 15 min under the same conditions. The pH was then adjusted to 7.0–7.4 using 0.1 m NaOH. Initial tests confirmed that the lowest crosslinker concentration yielded optimal gelling time. Aliquots of 200 µL were placed in 24‐well plates and UV‐crosslinked at 1 J/cm^2^. Hydrogels were purified by triple washing with PBS and ethanol to remove unbound Irgacure 2959 [13].

### 2.4 Characterization of Hydrogels

#### 2.4.1 Thermogravimetric Analysis (TGA)

Thermogravimetric analysis (TGA) was performed on ∼3 mg lyophilized hydrogel samples placed in ceramic trays. Samples were heated from 25°C to 600°C at 10°C/min under nitrogen atmosphere, recording mass changes and their rates.

#### 2.4.2 Differential Scanning Calorimetry (DSC)

Differential scanning calorimetry (DSC) was performed on ∼10 mg lyophilized GelMA samples placed in aluminum pans. Samples were analyzed under nitrogen atmosphere from −50°C to 300°C at 10°C/min, recording heat flux variations relative to air.

#### 2.4.3 Swelling Test

For swelling tests, 1.36 mL of 7% GelMA with 0.5% Irgacure 2959 was pipetted into petri dishes and crosslinked at 365 nm with 1 J/cm^2^. Hydrogels were then lyophilized, weighed (W_0_), and immersed in 2 mL water. Swelling was monitored by daily weights (W_1_) for 5 days after blotting. Equilibrium occurred when the weight stabilized [15]. Swelling ratios were calculated using the formula:

SwellingRatio(%)=(W1−W0)/W0×100

#### 2.4.4 Degradation Test

For enzymatic degradation tests, 1.36 mL of 7% GelMA with 0.5% Irgacure 2959 was pipetted into Petri dishes and crosslinked at 365 nm (1 J/cm^2^). Hydrogels were lyophilized in triplicate, weighed, and immersed in 2 mL PBS containing 2 U/mL collagenase type II at 37°C. The enzyme solution was withdrawn at 1, 3, 5, and 24 h; samples were stored at −80°C, re‐lyophilized, re‐weighed, and the weight loss calculated [16]:

DegradationRate(%)=((W0−Wt)/W0)×100

W_t_: weight of each GelMA hydrogel at time t

W_0_: initial weight of each GelMA hydrogel after lyophilization before enzyme addition

#### 2.4.5 Morphological Characterization

5% GelMA hydrogels were lyophilized to remove water, coated with gold‐palladium to enhance conductivity, and imaged by SEM at 450x magnification, 5 kV acceleration voltage, and 10 mm working distance. High‐resolution images were analyzed to evaluate surface morphology and microstructure [17].

#### 2.4.6 Rheology

Rheological properties were assessed using a rheometer per the manufacturer's protocol. A 20 mm parallel‐plate geometry was employed at 37°C. Dry gel samples were loaded onto the lower plate, with geometries set to a 700 µm gap and 0.1 N normal force. Frequency sweeps (0.01–10 Hz, 1% strain) determined storage modulus (G'), reflecting elasticity, and loss modulus (G''), reflecting viscosity [18].

#### 2.4.7 Fourier Transform Infrared Spectroscopy (FT‐IR)

FT‐IR spectra of lyophilized, uncrosslinked 5% and 7% (w/v) GelMA samples were recorded (4000–400 cm^−1^) using an FT‐IR spectrophotometer.

#### 2.4.8 1H‐NMR

GelMA solutions were prepared at 3%, 5%, and 7% (w/v) concentrations. Hydrogel samples underwent 1H‐NMR analysis.

### 2.5 Development of Vascular Bed in Hydrogel Designs

#### 2.5.1 Cell Culture

Human umbilical vein endothelial cells (HUVEC) were cultured in F‐12K medium supplemented with 10% fetal bovine serum (FBS) and 1% penicillin–streptomycin under standard conditions (37°C, 5% CO_2_). The culture medium was refreshed every 3 days to maintain optimal cell growth. For passaging, cells were detached using 0.25% trypsin–EDTA.

#### 2.5.2 Investigation of the Effect of Photoinitiator Concentration on HUVEC Viability in GelMA Hydrogels

GelMA precursor solutions with varying photoinitiator concentrations were mixed with HUVECs at 1 × 10^4^ cells/mL. Cell‐laden solutions were cast into 384‐well plates and photocrosslinked under UV light at 1 J/cm^2^. Post‐crosslinking, 50 µL complete medium was added per well, and constructs were incubated for 3 days under standard conditions. Cell viability was assessed via CellTiter‐Glo 3D assay: hydrogels were washed with PBS, 50 µL reagent added, protected from light, and incubated at room temperature for 25 min for lysis and signal stabilization. Luminescence was measured on a microplate reader to quantify viability.

#### 2.5.3 HUVEC Proliferation and Endothelial Tube Formation Assays

HUVEC proliferation was assessed over varying incubation periods using initial seeding densities of 1 × 10^3^ – 1.5 × 10^4^/well and VEGF concentrations of 1–40 ng/mL under standard conditions. Endothelial tube formation was evaluated via BME assay: 96‐well plates were coated with 50 µL BME/well, incubated at 37°C for 1 h to gel, seeded with HUVECs (1.5 × 10^3^/well) in medium + 10 ng/mL VEGF, and imaged by inverted microscopy over 24 h.

GelMA hydrogels loaded with VEGF, Ang‐1, and Ang‐2 were prepared to study HUVEC–hydrogel interactions. HUVECs were either encapsulated in the hydrogel precursor before gelation or seeded on preformed hydrogels at a density of 1.5 × 10^3^/well. After 3 days under standard culture conditions, viability was assessed by MTT assay and compared to controls to evaluate growth factor loading and seeding effects on HUVEC viability.

### 2.6 Gene and Protein Analysis

Expression of endothelial markers CD105 and VEGFR in HUVECs was assessed on tissue culture polystyrene and GelMA substrates via immunofluorescence. Cells were seeded at 1.5 × 10^3^/well in 96‐well plates on biosignal‐supplemented GelMA hydrogels and cultured for 3 days. Following fixation with paraformaldehyde, primary antibodies against CD105 and VEGFR were applied; after PBS washing, secondary antibodies were added, and samples were imaged using a live‐cell imaging system. Tissue culture–treated polystyrene (TCPS) and unsupplemented GelMA served as controls.

To evaluate endothelial marker expression at the transcriptional level, CD105 and VEGFR I/II mRNA levels were quantified by droplet digital PCR (ddPCR). HUVECs 1.5 × 10^3^/well) were seeded in 24‐well plates on biosignal‐supplemented GelMA hydrogels and cultured for 3 days. Total RNA was isolated using standard methods, reverse‐transcribed to cDNA (≥100 ng/µL RNA), and analyzed on a QX200 ddPCR system per the manufacturer's protocol.

### 2.7 Coculture of Cancer Associated Fibroblasts (CAF) and Macrophages

THP‐1 human monocytic cells were cultured in RPMI‐1640 medium supplemented with 10% FBS and 1% penicillin–streptomycin at 37°C and 5% CO_2_. Cell density was maintained by medium replenishment every 3 days. Differentiation of THP‐1 monocytes into macrophage‐like cells (M0) was induced by phorbol 12‐myristate 13‐acetate (PMA, 5–200 ng/mL) for 24–72 h, followed by culture in fresh complete medium [19, 20]. Cell adherence and morphology were monitored by optical microscopy. For M2 macrophage polarization, M0 macrophages were stimulated with macrophage colony‐stimulating factor (M‐CSF, 10 ng/mL) for 3 days [19].

Cancer‐associated fibroblast (CAF) cells were cultured in MSC‐GRO medium under standard conditions (37°C, 5% CO_2_). Cells between passages 1 and 10 were used. A stepwise oxygenation protocol was applied to allow adaptation to normoxic conditions without compromising cell morphology or proliferation.

CAF and M2 macrophages were co‐cultured at a 1:1 ratio in RPMI‐1640 medium supplemented with 2% FBS and 1% penicillin–streptomycin [21].

IL‐10 and TGF‐β protein levels were quantified by ELISA in monocytes, M0 and M2 macrophages, CAF‐M2 co‐cultures, and CAFs under 10% and 2% FBS conditions, per manufacturer's protocols. Lysates were prepared from 10^6^ cells/mL, total protein quantified by BCA assay, and results normalized to mg protein. IL‐10 and TGF‐β mRNA levels in M2 macrophages were assessed in 2% and 10% FBS media ± CAFs using ddPCR. Cells 1.5 × 10^4^/well) were seeded in 24‐well plates, incubated for 3 days, RNA isolated per protocol, reverse‐transcribed from ≥100 ng/µL RNA, and analyzed per ddPCR protocol.

### 2.8 Creating a Colon Cancer Model

The CRC drug screening model was established by seeding human colon cancer (Caco‐2) cells onto HUVEC‐laden hydrogels, accompanied by a binary CAF–macrophage co‐culture positioned on the TCPS surface beneath the hydrogel. Cellular functionality was optimally preserved over a 5 – 7 day period. Caco‐2 cells were maintained in high‐glucose DMEM supplemented with 10% FBS and 1% penicillin–streptomycin (37°C, 5% CO_2_) and seeded at 1.5 × 10^4^ cells/well for all experiments.

### 2.9 Investigating the Effects of 5‐Fluorouracil on The Model

#### 2.9.1 Cytotoxicity

Cell viability was assessed by MTT assay. Cells were exposed to increasing concentrations of 5‐FU (1, 5, 10, 25, 50 ve 100 µmm) for 24 and 72 h [22, 23]. Transwell inserts were removed where applicable, and cells were washed with PBS, detached using a gentle dissociation reagent (GCDR, Stemcell), and incubated with MTT solution at 37°C for 4 h. Plates were centrifuged, supernatants discarded, and formazan crystals solubilized in DMSO. Absorbance at 570 nm was measured on a microplate reader [24].

To compare the model with conventional 2D cultures, Caco‐2 cells were seeded at 1.5 × 10^4^ cells/well in 96‐well plates. Cells were treated with 5‐FU using the same concentrations and exposure times as in the model. Viability was then assessed by MTT assay [25].

Cell viability was expressed as a percentage relative to untreated control cells, and half‐ inhibitory concentration (IC_50_) values. Cells treated with drug‐free culture medium were used as negative controls.

#### 2.9.2 Reactive Oxygen Species (ROS) Production

Intracellular ROS levels were measured using the H_2_DCFDA assay. Cells were detached using GCDR, transferred to 96‐well plates, and centrifuged at 500 rpm for 18 min. Both hydrogel supported‐cultured cells and 2D‐cultured Caco‐2 cells were treated with the same drug concentrations as in cytotoxicity assays for 1 h at 37°C and 5% CO_2_. PBS, culture medium, and 100 µm H_2_O_2_ served as blank, negative, and positive controls, respectively. After treatment, cells were washed with PBS and incubated with H_2_DCFDA in the dark for 1 h at 37°C and 5% CO_2_. Wells were then washed with PBS, fresh PBS added, and fluorescence intensity (excitation/emission: 485/520 nm) measured. ROS levels were expressed relative to controls [24, 25].

#### 2.9.3 Genotoxicity

Alkaline comet assay (single‐cell gel electrophoresis) was used to assess genotoxicity. Cells were incubated for 3 h with 5‐FU concentrations below IC_50_ (from cytotoxicity assays), washed with PBS, and detached (GCDR for cells from the model; trypsin‐EDTA for 2D Caco‐2). Live cells were counted and diluted to 10^4^– 2 × 10^4^/slide. A 50 µL cell aliquot was mixed with 100 µL 1% LMA (37°C), spread on 1% NMA‐coated slides, and solidified. Slides were lysed at 4°C (1 h), equilibrated in electrophoresis buffer (20 min), electrophoresed (25 V, 300 mA; 20 min), neutralized in PBS (15 min), dehydrated in ethanol series (50%, 75%, 99%; 5 min each), and stained with EtBr. Tail moments were quantified for 100 cells/slide using Comet Assay IV software under fluorescence microscopy [25].

#### 2.9.4 Apoptosis

Colorectal cancer cells in the model and 2D‐cultured Caco‐2 cells were incubated for 24 h with 5‐FU solutions at concentrations close to the IC_50_ values determined by cytotoxicity assessments. Subsequently, cells from the model were collected using GCDR, while 2D‐cultured Caco‐2 cells were detached using trypsin‐EDTA solution. The cells were then stained with the Annexin V‐FITC kit and propidium iodide and analyzed by flow cytometry [24, 25].

#### 2.9.5 Statistical Analysis

Data analysis was performed using SPSS 23.0. Normality of data was assessed with the Kolmogorov–Smirnov test and homogeneity of variances with Levene's test. Normally distributed data was analyzed using Student's t‐test (two groups) or ANOVA with LSD post‐hoc test (multiple groups). Non‐normally distributed data were analyzed using Mann–Whitney U test (two groups) or Kruskal–Wallis test (multiple groups). Experiments were conducted in triplicate; results are mean ± SD. Statistical significance was set at *p* < 0.05.

## 3 Results and Discussion

### 3.1 Hydrogel Preparation

**FIGURE 1:**
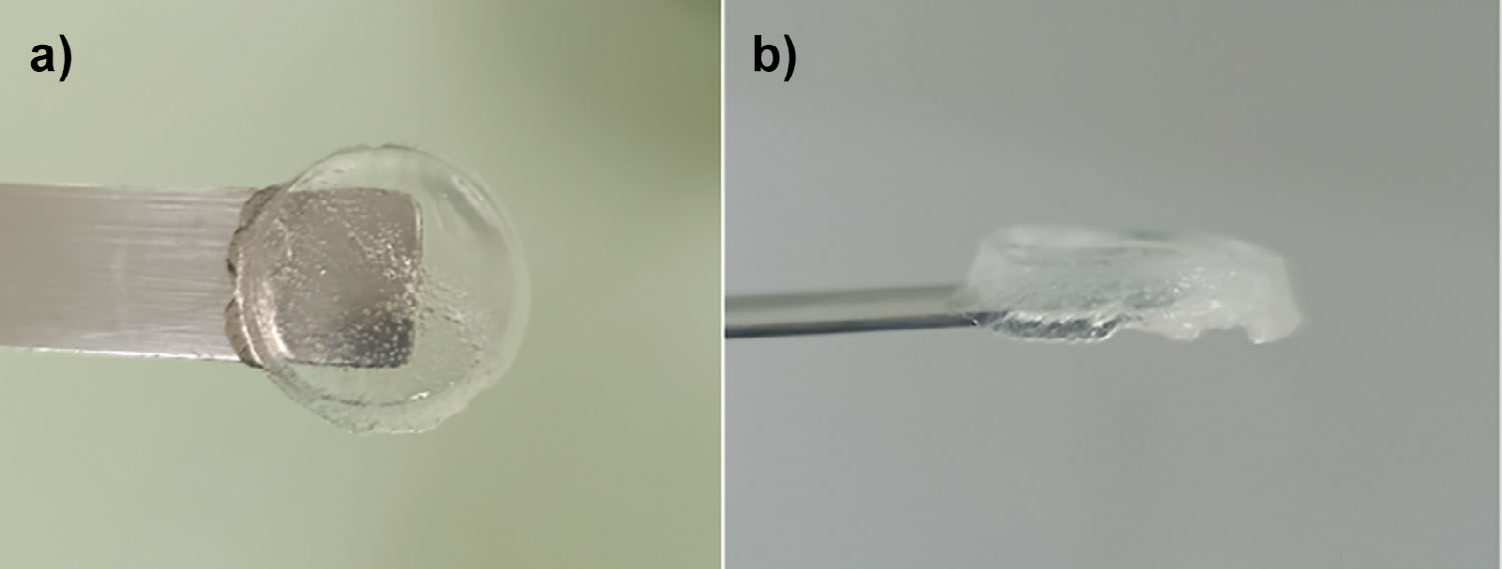
(a) Top and (b) Side views of 7% GELMA hydrogels prepared in a 48‐well cell plate after UV cross‐linking.

GelMA hydrogel preparation commenced by dissolving GelMA in PBS [26] optimizing a gelling protocol tailored for tissue engineering under controlled conditions, as inspired by established methodologies. Comprehensive literature reviews and empirical observations confirmed that optimal gelling times are reliably achieved with at least 0.5% crosslinking agent [27], while lower concentrations failed to induce crosslinking under identical UV exposure. Precisely weighed GelMA was dissolved in the requisite PBS volume and stirred vigorously for 30 min at 37°C using a heated magnetic stirrer. Subsequently, Irgacure 2959 crosslinker followed by an additional 30‐min stir at 37°C in darkness to prevent premature activation. For seamless cell culture compatibility, pH was meticulously adjusted to physiological levels (∼7.4). The finalized solutions were dispensed into 96‐well and 48‐well plates, yielding an optimized GelMA based on growth areas of 0.33 and 0.84 cm^2^, respectively. This standardization enabled robust characterizations and cell studies across varying GelMA densities per unit area. Uncured solutions were pre‐incubated in darkness at room temperature for 10 min to initiate gelation, then exposed to 365 nm UV light for 2 min to fully activate crosslinking, consistently yielding stable, transparent hydrogels with excellent shape retention (Figure 1).

To achieve robust, cross‐linked GelMA hydrogels that reliably retain their structural integrity and shape across all wells following UV exposure, comprehensive stability assessments were essential. Formulations at 3% w/w GelMA proved critically deficient, exhibiting rapid disintegration that precluded accurate measurement in swelling and degradation analyses‐necessitating their exclusion from all characterization studies [28]. In tissue engineering applications, aqueous stability is non‐negotiable; the 3% GelMA's vulnerability stems from sparse methacrylamide crosslinking, which fosters expansive gaps between polymer chains, accelerates water ingress, weakens methacrylate bonds, and hastens degradation due to minimal gelatin substrate. Thus, these underperforming gels were decisively omitted to ensure the reliability of subsequent evaluations.

### 3.2 Characterization of Hydrogels

**FIGURE 2:**
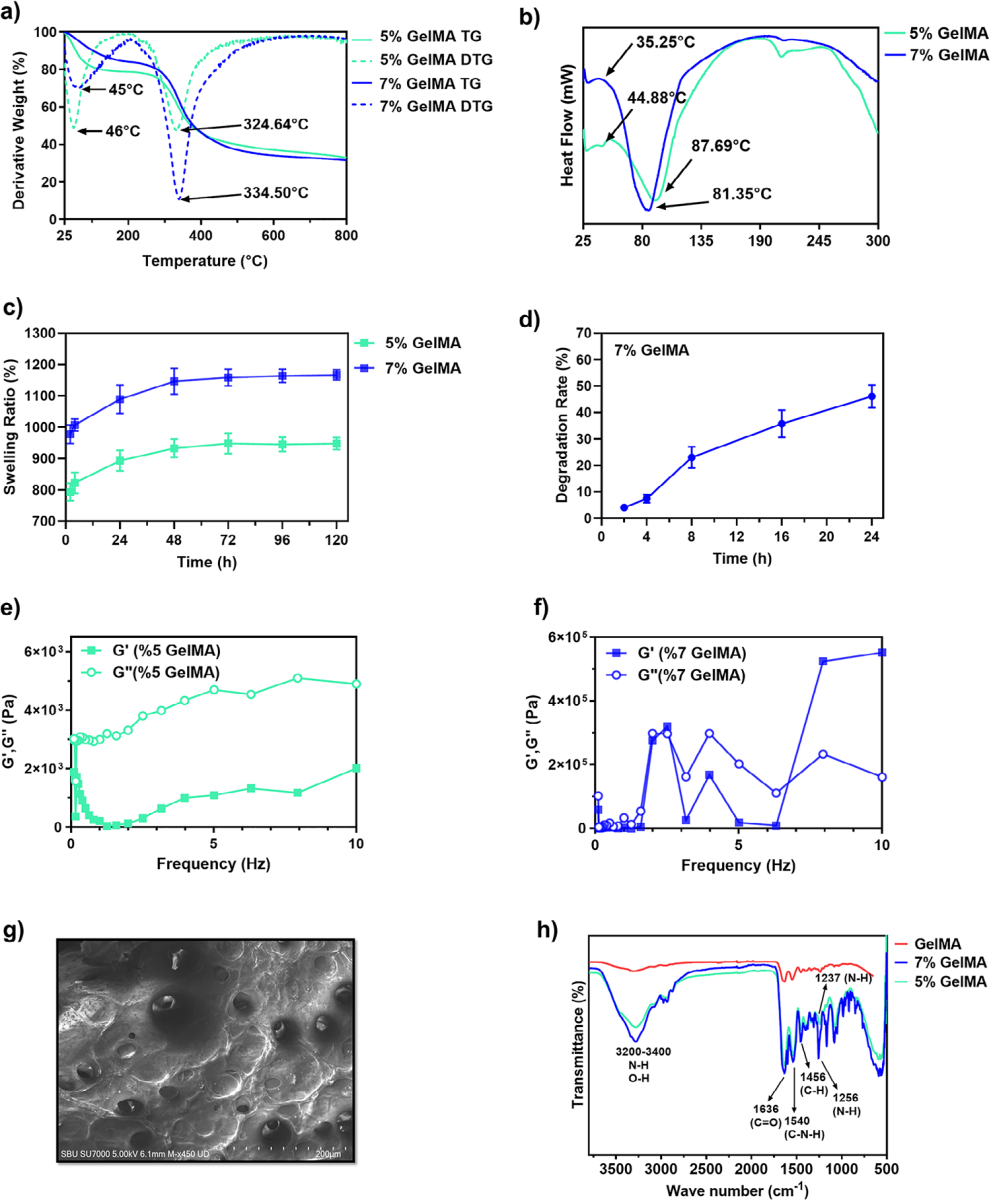
Physicochemical, thermal, structural, and rheological characterization of GelMA hydrogels; (a) Thermogravimetric analysis (TGA) of GelMA hydrogels at 5% and 7% concentrations, (b) Differential scanning calorimetry (DSC) thermograms of GelMA hydrogels, (c) Swelling behavior of GelMA hydrogels expressed as swelling ratio, (d) Enzymatic degradation profile of GelMA hydrogels monitored for 24 h, presented as percentage dry weight loss, requency‐dependent rheological behavior of (e) 5% and (f) 7% GelMA hydrogels, illustrating storage modulus (G′) and loss modulus (G″) variations, (g) Scanning electron microscopy (SEM) analysis of gels (Scale 200 µm), (h) Fourier‐transform infrared (FTIR) spectra of GelMA hydrogels.

When the TGA results of the obtained hydrogels were examined, mass losses occurred at different temperatures for both 5% and 7% concentrations of GelMA hydrogels. The first mass loss occurred between 45°C and 50°C, and this was attributed to the evaporation of water in the hydrogel structure [29]. It was also observed that mass loss occurred at different temperatures depending on the different GelMA concentrations. For example, the peak temperature of mass loss in hydrogels containing 5% GelMA was measured as 324.64°C, while this temperature increased to 334.50°C at the 7% concentration (Figure 2a). As a result of examining the thermal properties of the prepared GelMA hydrogels using DSC, it was determined that there were significant differences in the glass transition temperatures (Tg) of the samples prepared at different concentrations [30]. The Tg value was measured as 44.88°C for GelMA samples prepared at a concentration of 5% by volume weight, while the Tg value was determined as a lower temperature of 35.25°C in the samples prepared at a concentration of 7%. The denaturation temperature was determined as 87.69°C in GelMA samples prepared at a concentration of 5%, while this temperature was measured as 81.35°C in the samples prepared at a concentration of 7% (Figure 2b).

To assess the stability of the hydrogels in an aqueous environment, swelling analyses were performed over 144 h, revealing a swelling ratio of 18.91 ± 0.10 (Figure 2c). In parallel, degradation studies monitored enzymatic distribution, with hydrogel behavior tracked for up to 24 h; at the end of this period, the structures lost 1.62 ± 1.44 of their dry weight (Figure 2d).

Rheological analysis of 5% GelMA reveals that as frequency increases, the loss modulus (G'') values remain higher than the storage modulus (G') values. Nevertheless, G' shows a significant increasing trend across the frequency range, starting from a low initial value and exhibiting a gradual rise with increasing frequency. In the rheological analysis of 7% GelMA hydrogel, moreover, the storage modulus (G') values increase significantly as frequency rises; this trend indicates enhanced mechanical stability of the hydrogel and strengthened structural resistance at high frequencies. In contrast, the loss modulus (G'') values are lower than G', albeit showing variability in certain frequency ranges (Figure 2e,f).

As seen in Figure 2g, SEM analysis of GelMA hydrogels revealed a highly porous microstructure with significantly large surface pores, providing insights into the microscopic network connections between polymeric chains. Cross‐linked polymer networks regulate mechanical properties in biological systems, such as cellular microenvironments, by controlling the material's physical properties [31].

FTIR analysis was used to identify and verify the functional groups present in the chemical structure of GelMA samples. The broad band observed in the 3200–3400 cm^−1^ range in the spectrum indicates the presence of peptide bonds, reflecting the stretching vibrations of amide (N─H) and hydroxyl (O─H) groups [32]. The peak at 1636 cm^−1^ represents the carbonyl (C═O) stretching vibrations of the amide I groups, while the peak at 1540 cm^−1^ shows the vibrations associated with C─N stretching and N─H bending of the amide II region. These peaks clearly reveal the protein‐based chemical structure of GelMA [33]. The peak at 1456 cm^−1^ indicates C─H deformation vibrations of methacrylic groups, while distinct peaks of N─H bending vibrations were detected at 1256 and 1237 cm^−1^ (Figure 2h) [34].

NMR spectroscopy is widely used to verify the chemical modifications of gelatin and GelMA and to determine the degree of methacrylation. NMR analysis provides critical information to examine the changes occurring in the molecular structure of gelatin in detail and to confirm the successful synthesis of GelMA [35]. Therefore, ^1^H NMR analyses were performed on three different lyophilized GelMA hydrogel, gelatin, and uncrosslinked GelMA samples at concentrations of 3%, 5%, and 7% by weight. When the NMR spectra of gelatin and GelMA are examined, characteristic changes in the spectral properties of gelatin are observed as a result of methacrylation. First, when comparing the spectra of gelatin and GelMA (SI 1), distinct peaks were observed in the NMR spectrum of GelMA in the range of approximately 5.3–5.6 ppm. These peaks correspond to the acrylic protons of the methacrylate groups and indicate that the methacrylate groups were successfully grafted onto the gelatin backbone [36]. In addition, the signal at approximately 1.8 ppm was attributed to the methyl protons of the grafted methacrylate group. This signal is not found in pure gelatin and is a unique indicator of methacrylation [37]. During the attachment of methacrylate groups to the lysine and hydroxylysine residues of gelatin, it was observed that the peaks associated with lysine‐NH_2_ (2.8–3.0 ppm) weakened or disappeared [38]. This confirms that methacrylate anhydride is covalently bonded to the free amino groups of gelatin. In addition, the phenylalanine signal in the range of 7.1–7.4 ppm is observed in both gelatin and GelMA [39]. NMR analyses also show the emergence of new functional groups in the gelatin backbone during the modification process. New peaks in the range of 5.4 and 5.6 ppm directly support the binding of methacrylate groups to gelatin, while the decrease or disappearance of the lysine methylene signal at 3.0 ppm confirms the transformation of free amino groups of gelatin [35] Supporting Information.

### 3.3 Development of Vascular Bed in Hydrogel Designs

**FIGURE 3:**
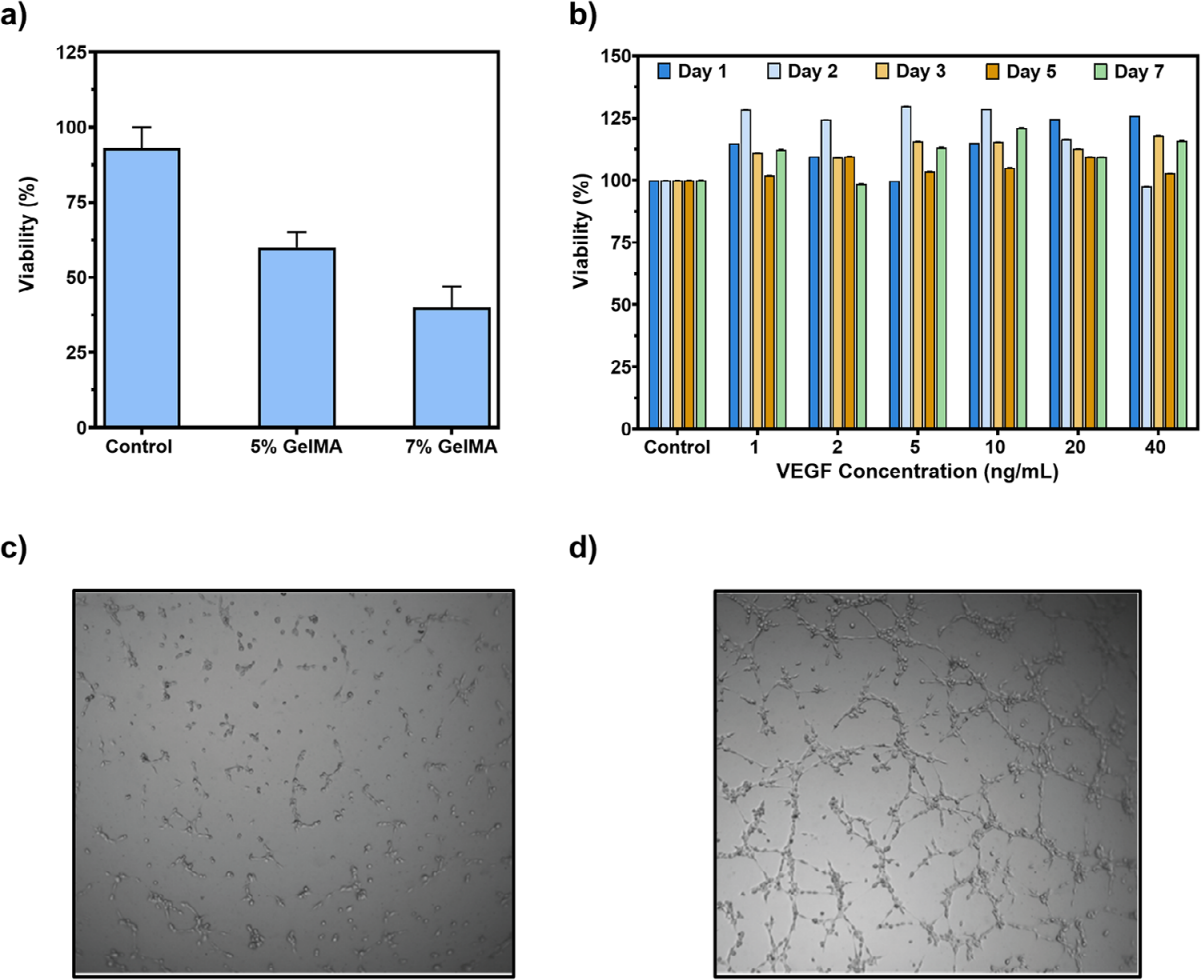
Cytocompatibility and pro‐angiogenic effects of GelMA hydrogels and VEGF on HUVEC cells; (a) Cell viability of HUVEC cells cultured in the presence of GelMA hydrogels prepared at different concentrations, (b) Effect of varying VEGF concentrations (1–40 ng/mL) on the viability of HUVEC cells cultured in 7% GelMA‐containing medium over different incubation periods (days 1, 2, 3, 5, and 7), (c) Representative microscopic images of HUVEC cells cultured in complete medium supplemented without VEGF after 24 h of incubation (Scale 500 µm), (d) Representative microscopic images of endothelial tube formation by HUVEC cells cultured in complete medium supplemented with VEGF after 24 h of incubation (Scale 500 µm).

The MTT test was used to evaluate the interactions of cells with GelMA hydrogels prepared at different concentrations. The results showed a decrease in cell viability for all GelMA concentrations. However, the loss of viability in cells treated with 5% GelMA was observed to be 15% lower compared to the 7% GelMA group, and this difference was not statistically significant (Figure 3a). When the effects of different VEGF concentrations (1–40 ng/mL) on the viability of HUVEC cells in a medium containing 7% GelMA were examined over different incubation periods (days 1, 2, 3, 5, and 7), it was observed that the effect of VEGF on cell viability tended to decrease over time (Figure 3b). When the effects of VEGF on endothelial tube formation were examined following its effects on cytotoxicity, as shown in Figure 3c,d, it was observed that HUVEC cells cultured in complete culture medium supplemented with VEGF exhibited endothelial tube formation after 24 h of incubation following plating. This finding underscores the pro‐angiogenic capacity of VEGF within the GelMA matrix, demonstrating its crucial role in promoting the organization of endothelial cells into capillary‐like structures. Additionally, the spatial distribution of matrix‐bound and soluble growth factors, particularly VEGF, significantly influences tumor progression and angiogenesis within these biomimetic systems [40]. Although 7% GelMA alone reduced HUVEC viability, VEGF supplementation substantially improved cell survival even at the lowest concentration tested. The lack of a strictly dose‐dependent response among VEGF‐treated groups suggests that the endothelial response may have reached a threshold/saturation level within the investigated concentration range. Determination of the lower effective VEGF concentration limit will require evaluation of sub‐threshold doses in future studies.

**FIGURE 4:**
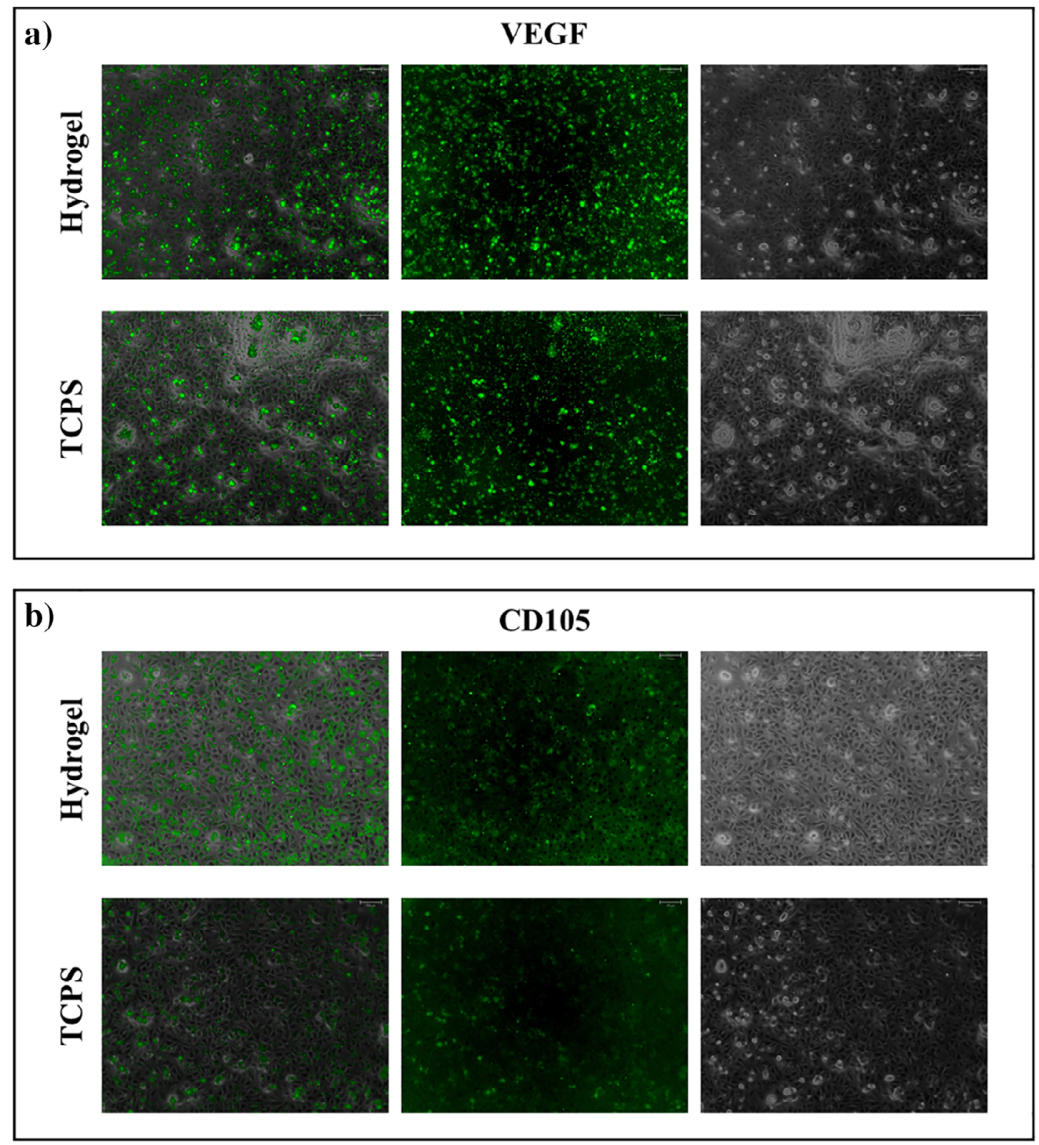
Immunofluorescence analysis of endothelial markers in HUVEC cells cultured within GelMA hydrogels (Scale 500 µm).

HUVEC cells were seeded into the GelMA hydrogel and stained for CD105 and VEGF markers. As shown in Figure 4, live cells were visualized in green using CellBrite staining to assess their viability and distribution within the matrix. Compared to the TCPS control, GelMA‐cultured cells exhibited noticeably higher fluorescence signals for both CD105 and VEGF. These findings indicate that the GelMA hydrogel provides a more favorable microenvironment for endothelial cell activity and the expression of angiogenic markers. Nevertheless, as these observations are based on qualitative fluorescence imaging, they should be interpreted with appropriate caution [41].

### 3.4 Gene and Protein Analysis

**FIGURE 5:**
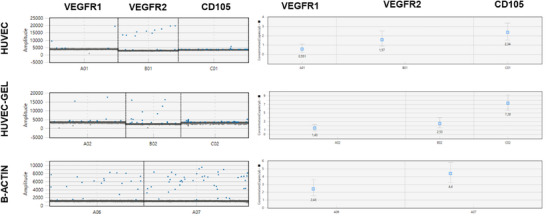
Demonstration of VEGF I/II and CD105 gene expression in HUVEC cells in GelMA hydrogel and on TCPS surfaces.

The behavior of HUVEC cells in growth factor‐doped GelMA hydrogels was also analyzed at the gene level, specifically focusing on the expression levels of VEGFR1/2 and CD105 genes. In this regard, digital droplet PCR analysis of both free HUVEC cells and those embedded in growth factor‐enriched GelMA hydrogels revealed successful droplet formation. While an average of 6000–8000 droplets were analyzed for free HUVEC cells, this number increased to up to 10 000 in the gel‐embedded samples. Consequently, this indicates the presence of gene populations that generate higher signal intensities following gel incorporation. After normalization to β‐actin, cells cultured in GelMA exhibited 2.92‐fold and 5.65‐fold higher expression of VEGFR2 and CD105, respectively (Figure 5). These elevated expression levels suggest that the GelMA hydrogel microenvironment significantly upregulates key receptors and markers associated with endothelial cell activation and angiogenesis, promoting a more robust angiogenic response than observed in free HUVEC cells [42, 43].

### 3.5 Coculture of Cancer Associated Fibroblasts (CAF) and Macrophages

**FIGURE 6:**
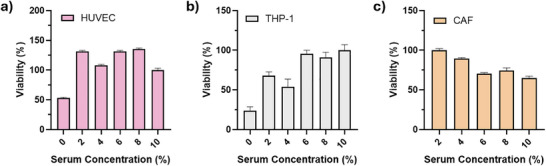
Effect of serum concentration on the viability of HUVEC, THP‐1, and CAF cell lines.

HUVEC, THP‐1, and CAF cell lines were seeded into 96‐well plates and cultured with serum concentrations ranging from 0% to 10%. Cell viability was quantitatively assessed using the MTT assay once approximately 70% proliferation was achieved at standard serum concentrations. For THP‐1 cells, differentiation was induced under appropriate medium conditions, followed by MTT analysis two days after PMA treatment. The effects of serum concentration on proliferation varied by cell type. HUVEC cells reached peak rates at 2–6% serum, declining thereafter at 8% and 10%. In contrast, CAF cells progressively lost proliferation with increasing serum, hitting lowest rates at 10%, while THP‐1 cells remained stable at low levels but dropped significantly beyond 6%. These results show HUVEC cells thrive at moderate serum, whereas CAF and THP‐1 are inhibited by high levels. Thus, a 4–6% serum range optimizes proliferation across all lines, maximizing HUVEC growth with minimal impact on the others. This careful calibration of serum concentration is critical for establishing an in vitro model that accurately mimics the complex tumor microenvironment, where diverse cell types interact under specific nutrient conditions. This optimized serum concentration regimen is crucial for maintaining the physiological relevance of the biomimetic platform, allowing for the accurate assessment of drug efficacy and cellular responses within a controlled microenvironment. Such precise environmental control is essential for investigating the intricate interplay between various cell types and drug compounds, ultimately enhancing the predictive power of the in vitro model for cancer drug screening (Figure 6).

**FIGURE 7:**
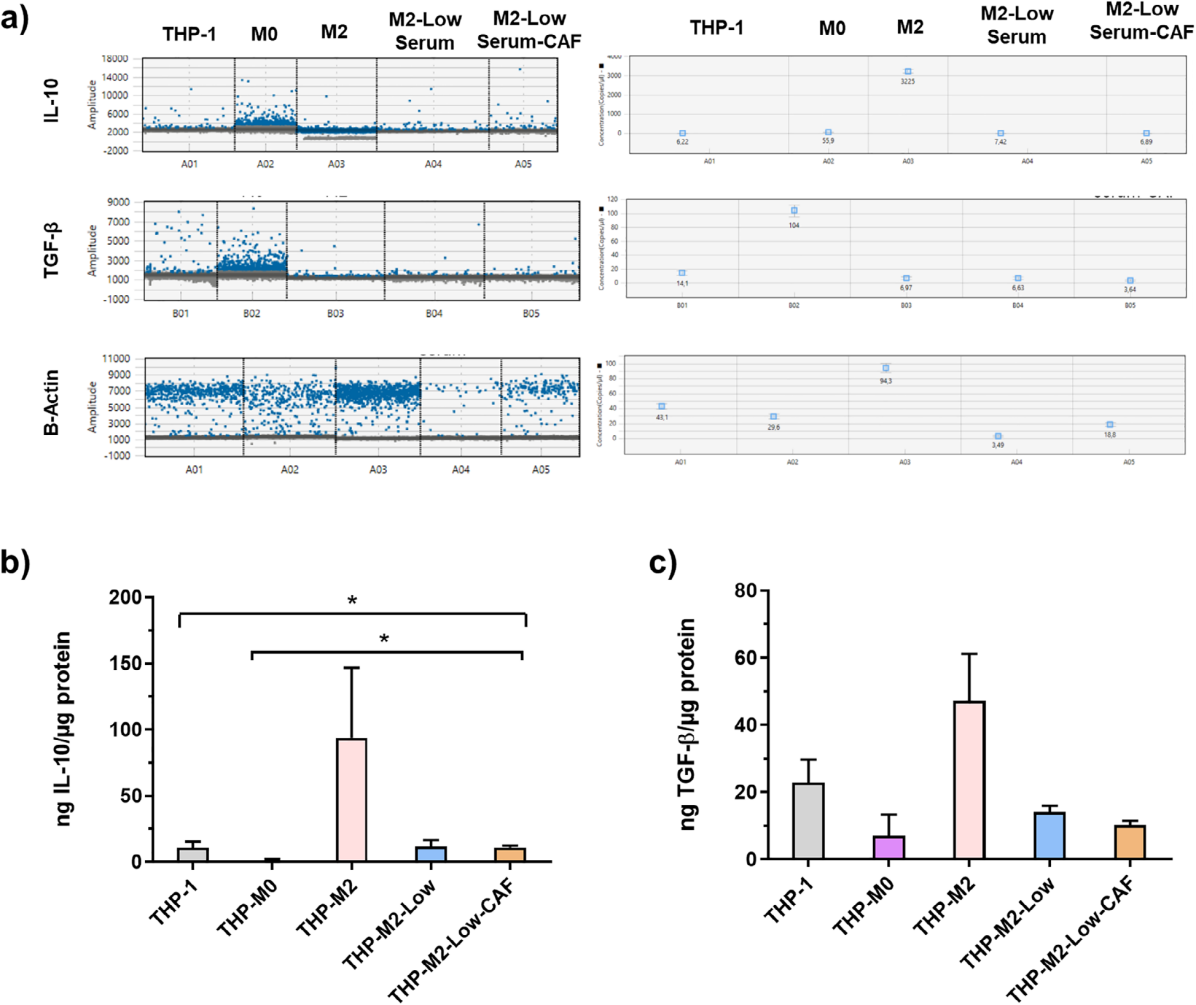
Effects of serum conditions and CAF co‐culture on IL‐10 and TGF‐β expression in macrophages; (a) Relative IL‐10, TGF‐β, and β‐actin gene expression levels in M0 and M2‐polarized macrophages cultured under standard and low serum conditions, with or without cancer‐associated fibroblast (CAF) co‐culture, (b) Protein‐level quantification of IL‐10 in macrophage culture supernatants, (c) Protein‐level quantification of TGF‐β in macrophage culture supernatants.

Changes in IL‐10 and TGF‐β levels in M2‐polarized macrophages under low serum concentrations and in the presence of CAF were monitored at both gene and protein levels. As expected, M2 polarization under standard medium conditions increased IL‐10 levels while decreasing TGF‐β levels. However, low serum concentrations combined with CAF inclusion reversed the IL‐10 increase and further reduced TGF‐β levels. In addition to gene expression analyses, ELISA assays were performed on the corresponding proteins. Consistent with the gene data, IL‐10 protein levels were higher under normal culture conditions compared to other groups, although this difference was not statistically significant. Conversely, IL‐10 protein levels increased significantly in M0 macrophages in the presence of CAF (Figure 7a–c). This suggests that CAF exerts a significant influence on macrophage phenotype, potentially driving an immunosuppressive microenvironment conducive to tumor progression, as evidenced by the upregulation of IL‐10, a cytokine known to promote tumor growth and poor prognosis [44]. Conversely, elevated TGF‐β levels, especially in co‐culture with CAF, corroborate the immunosuppressive environment, fostering tumor progression through mechanisms such as T‐cell anergy and enhanced extracellular matrix deposition [45]. The interaction between CAF and M2 macrophages thus appears to be a critical determinant of the immunomodulatory landscape within the tumor microenvironment, with implications for therapeutic strategies targeting these cellular components [44].

### 3.6 Creating a Colon Cancer Model

**FIGURE 8:**
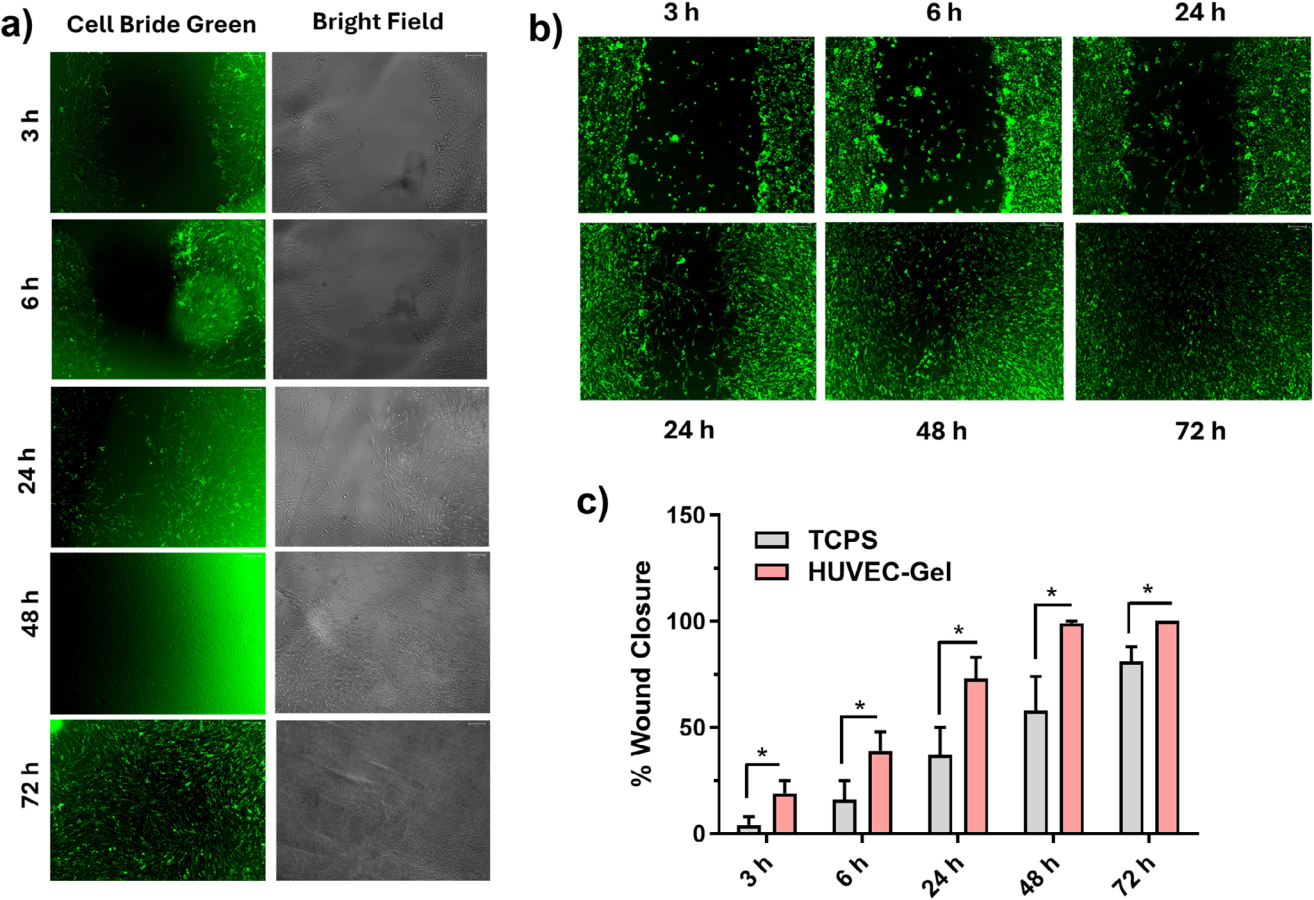
Migration capabilities of HUVEC cells on (a) GelMA and (b)TCPS at low serum concentration (2%) using green fluorescent membrane dye. Scale 500 µm, (c) Migration rate of HUVEC cells, ^*^significant difference between groups (*p*<0.05).

Under low serum conditions, HUVEC cells exhibited significantly higher migration rates on the GelMA hydrogel compared to the TCPS surface. Notably, cells on the GelMA completely closed the scratch wound within 48 h, whereas those on TCPS required 72 h for full closure (Figure 8). This enhanced migratory capacity on GelMA suggests that the hydrogel matrix, even under nutrient‐limited conditions, provides a more permissive environment for endothelial cell movement, a crucial process in angiogenesis and wound healing. The accelerated migration on GelMA underscores its potential for tissue engineering applications requiring rapid endothelialization and vascular network formation, even when nutrient availability is restricted. The more pronounced visual change observed at the 48 h time point in the TCPS group likely reflects a transitional phase of collective cell migration under low‐serum conditions. At this intermediate stage, cells at the wound edge exhibit increased spreading and temporary reorganization, leading to a more distinct fluorescence pattern. As migration progresses, this organized front becomes less visually prominent at later time points (72 h) due to further cell redistribution and partial wound closure. Therefore, the observed difference at 48 h represents a dynamic stage of the migration process rather than a discrete or isolated biological effect.

### 3.7 Investigating the Effects of 5‐Fluorouracil on the Model

Following the establishment of a sophisticated CRC model using Caco‐2 cells, the potent effects of 5‐FU ‐a proven frontline therapy against colon cancer‐ on cytotoxicity, genotoxicity, ROS release, and apoptosis were meticulously evaluated across both 2D and 3D cultures. Comparative analysis of these results compellingly demonstrates the model's superior utility as a reliable, high‐fidelity drug screening platform.

**FIGURE 9:**
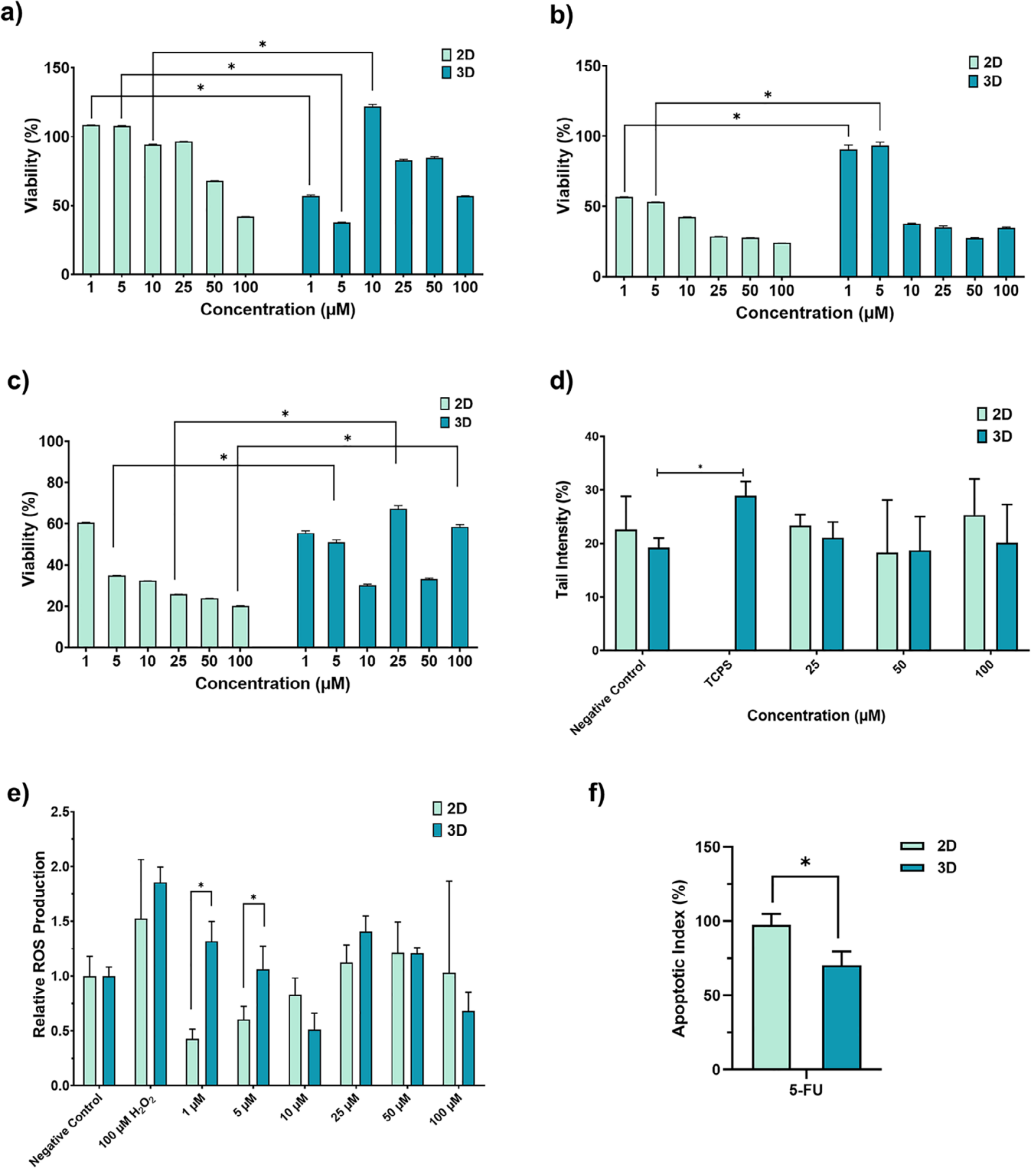
Comparative evaluation of 5‐fluorouracil (5‐FU) responses in 2D and 3D CRC models; (a) Cytotoxicity analysis of 5‐FU in colorectal cancer cells cultured under 2D and 3D conditions at different drug concentrations after (a) 24 h, (b) 48 h, (c) 72 h, (d) Genotoxicity assessment of 5‐FU using the Comet assay in 2D and 3D models, (e) Relative reactive oxygen species (ROS) generation in 2D and 3D colorectal cancer cells following treatment with increasing concentrations of 5‐FU, (f) Apoptosis analysis in 2D and 3D colorectal cancer cells following 5‐FU treatment.

Examination of the results revealed significant differences in cytotoxic effects between 2D and 3D cultures, particularly at low drug concentrations; however, these differences were not observed at higher concentrations. Moreover, the cytotoxic disparities became more pronounced with increasing drug exposure time (Figure 9a–c). The observed discrepancies in drug response underscore the critical role of 3D microenvironments in modulating chemotherapeutic efficacy, highlighting the limitations of traditional 2D assays in predicting in vivo outcomes. This divergence emphasizes the importance of incorporating complex cellular interactions and spatial organization, such as those mimicked in 3D models, for accurate drug screening and the development of novel therapeutic strategies [46].

As shown in Figure 9d, although 5‐FU at low doses reduced ROS levels compared to the negative control, an increase in ROT levels was observed with increasing dose. Furthermore, the increase in ROS levels was more pronounced in 3D cells compared to the negative control. This suggests that 3D cell cultures might exhibit heightened sensitivity to oxidative stress induced by 5‐FU, potentially due to altered metabolic states or protective mechanisms within the more complex architecture [47]. This differential response highlights the importance of employing 3D models to accurately assess drug‐induced oxidative stress and predict patient responses [48].

Given the potential for DNA damage to induce lethal effects in cancer cells, the genotoxic impact of 5‐FU on 2D and 3D CRC cells was evaluated using the Comet assay. Compared to the negative control, 5‐FU did not cause genotoxic effects in either culture type. Although the gel used in the 3D model induced some DNA damage relative to the control, this did not influence the drug screening system's response, with no differences observed between 2D and 3D cells (Figure 9e). These findings suggest that while the 3D scaffold might intrinsically cause a low level of DNA damage, it does not impede the assessment of 5‐FU's genotoxicity, affirming the model's robustness for drug screening. Conversely, 3D cultures consistently demonstrated lower sensitivity to various chemotherapeutic agents, including 5‐FU, doxorubicin, and cisplatin, often requiring higher concentrations to achieve comparable cytotoxic effects observed in 2D cultures [49].

Studies in CRC cells have shown that the active ingredient 5‐FU at a concentration of 100 µM can exert its effect by inducing apoptosis in CRC [50]. In this study, it was observed that these drugs induced apoptosis in both 2B and 3B cells, although the increase in apoptosis was more pronounced in 2D cells. However, a significant difference was found in the apoptotic index findings obtained in the 2D and 3D CRC models (Figure 9f).

## 4 Conclusion

In this work, we present a GelMA‐based, multicellular 3D *in vitro* CRC platform that bridges biomaterials engineering with functional drug screening applications. By integrating endothelialized hydrogel matrices with stromal and immune cell components, the system enables the reconstruction of essential tumor microenvironmental features that are absent in conventional 2D culture models. Comprehensive material characterization confirmed that the physicochemical and mechanical properties of GelMA hydrogels directly influence endothelial behavior and vascular‐associated signaling, underscoring the importance of material‐driven regulation of biological function. When applied to chemotherapeutic evaluation, the platform revealed markedly different drug response profiles compared to 2D cultures, including reduced cytotoxicity, oxidative stress, and apoptosis following 5‐FU exposure. These differences highlight the critical role of multicellular crosstalk and mass transport limitations in governing therapeutic efficacy. Collectively, the results demonstrate that biomaterials‐engineered 3D microenvironments provide more physiologically relevant and predictive drug screening outcomes than traditional monolayer systems. The proposed platform offers a robust and versatile framework for preclinical anticancer drug evaluation and supports the development of human‐relevant testing strategies that may reduce reliance on animal models. With further validation across additional drug classes and disease contexts, this system holds strong potential as a translational tool in biomaterial‐driven cancer research Supporting Information.

## Author Contributions

**Seyfure Adıgüzel**: Formal analysis, Validation, Data curation, Visualization; **Sevde Altuntaş**: Methodology, Formal analysis, Validation, Data curation, Writing – Review & Editing; Visualization; **Şevval Çelikten**: Data curation, Formal analysis; Mehmet Cem Gündüz: Data curation, Formal analysis; **Merve Güdül Bacanlı**: Conceptualization, Methodology, Project administration; Supervision, Funding acquisition, Formal analysis, Validation, Data curation, Writing – Original Draft, Writing – Review & Editing; Visualization.

## Conflicts of Interest

The authors declare no conflicts of interest.

## Supporting information




**Supporting File**: mabi70190‐sup‐0001‐SuppMat.docx.

## Data Availability

The data that support the findings of this study are available from the corresponding author upon reasonable request.
