# Supplementary material for: A Multicellular 3D GelMA‐Based Colorectal Cancer Model for Chemotherapeutic Responses
Source: Macromol Biosci. 2026 May 1;26:e70190. doi: 10.1002/mabi.70190 (PMC13134817; doi:10.1002/mabi.70190)
Supplement: Supplementary file 1 — Supporting File: mabi70190‐sup‐0001‐SuppMat.docx. [file MABI-26-e70190-s001.docx]

**SUPPORTING INFORMATION**

**SI 1. a)**


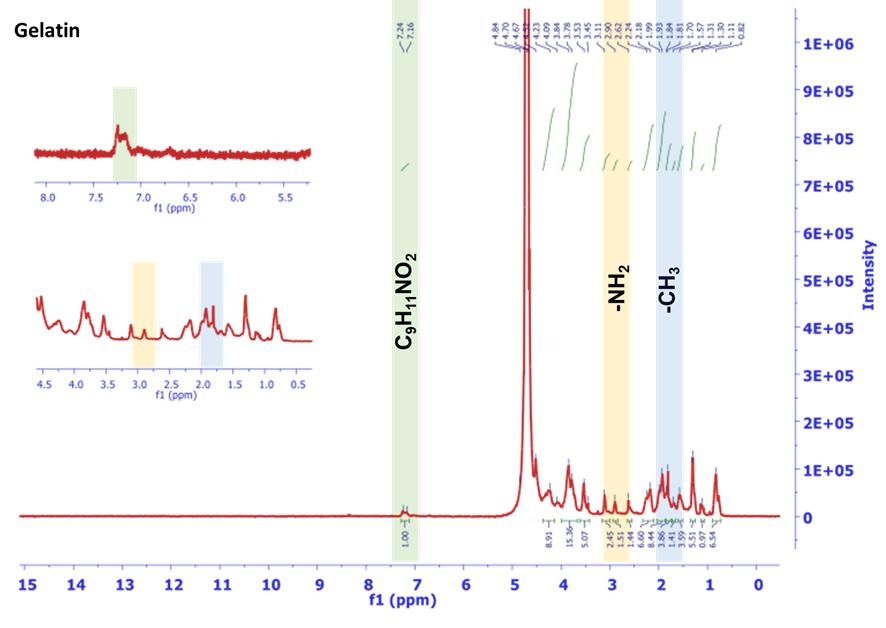


**b)**


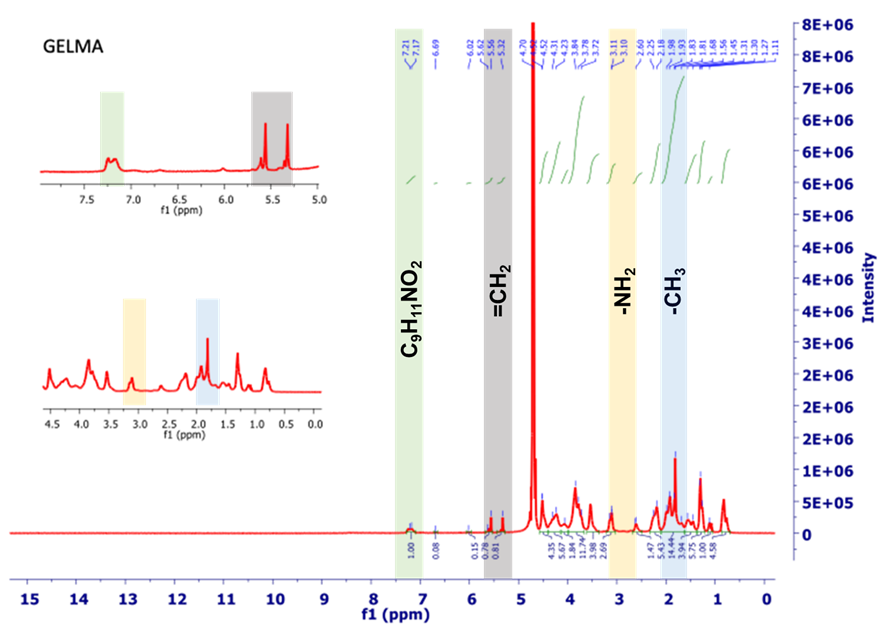


NMR spectra of a) Gelatin and b) Uncrosslinked GelMA

**SI 2. a)**


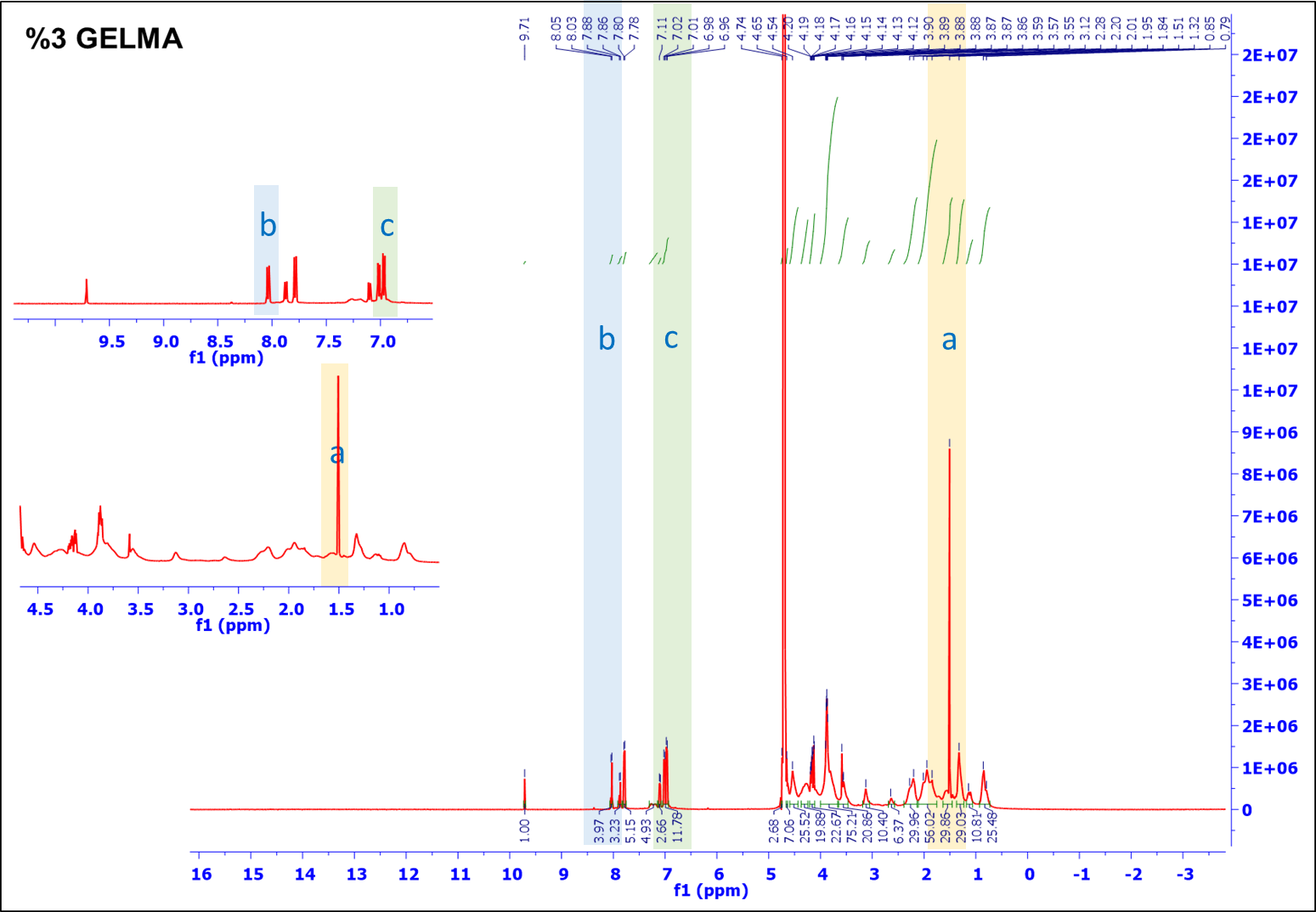


**b)**


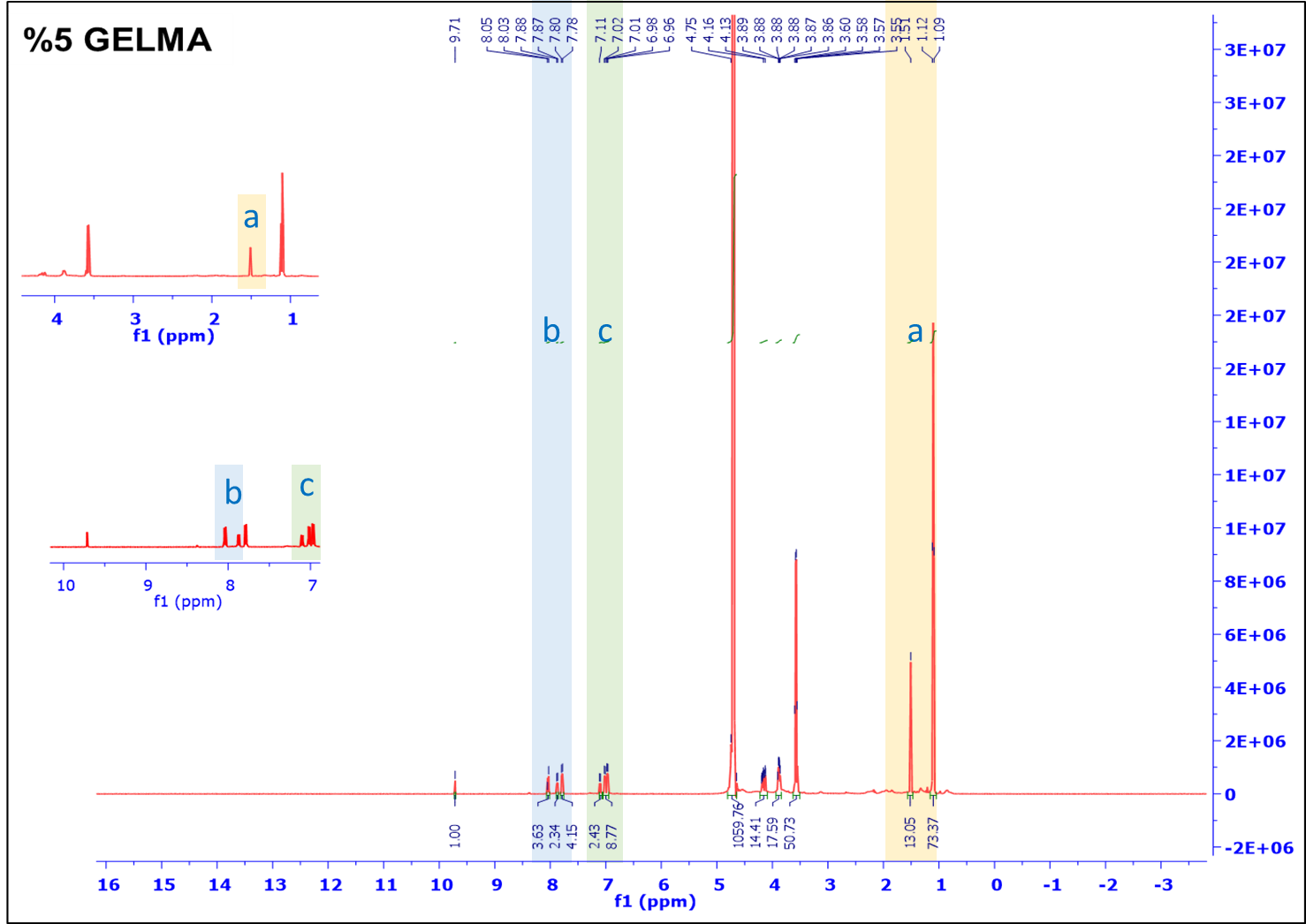


**c)**


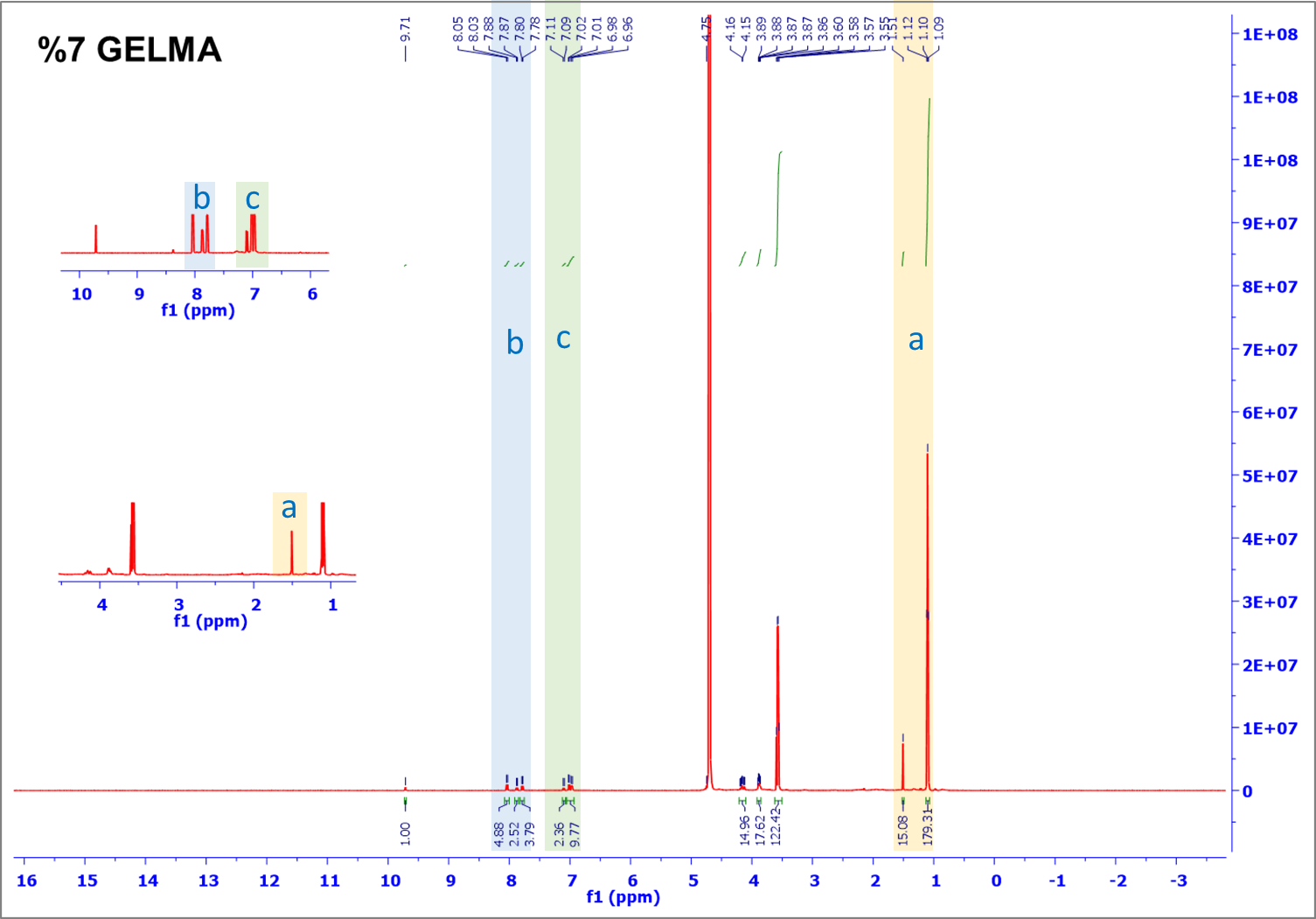


NMR spectra of a) 3% (w/v) GelMA, b) 5% (w/v) GelMA, c) 7% (w/v) GelMA hydrogels.
